# De novo full length transcriptome analysis of a naturally caffeine-free tea plant reveals specificity in secondary metabolic regulation

**DOI:** 10.1038/s41598-023-32435-5

**Published:** 2023-04-12

**Authors:** Xiaozeng Mi, Chun Yang, Dahe Qiao, Mengsha Tang, Yan Guo, Sihui Liang, Yan Li, Zhengwu Chen, Juan Chen

**Affiliations:** Tea Research Institute, Guizhou Academy of Agricultural Sciences, 1 Jin’nong Road, Guiyang, 550006 Guizhou China

**Keywords:** Chemical biology, Molecular biology

## Abstract

Tea plants are crops with economic, health and cultural value. Catechin, caffeine and theanine are the main secondary metabolites of taste. In the process of germplasm collection, we found a resource in the Sandu Aquatic Autonomous County of Guizhou (SDT) that possessed significantly different characteristic metabolites compared with the cultivar ‘Qiancha 1’. SDT is rich in theobromine and theophylline, possesses low levels of (−)-epicatechin-3-gallate, (−)-epigallocatechin-3-gallate, and theanine content, and is almost free of caffeine. However, research on this tea resource is limited. Full-length transcriptome analysis was performed to investigate the transcriptome and gene expression of these metabolites. In total, 78,809 unique transcripts were obtained, of which 65,263 were complete coding sequences. RNA-seq revealed 3415 differentially expressed transcripts in the tender leaves of ‘Qiancha 1’ and ‘SDT’. Furthermore, 2665, 6231, and 2687 differentially expressed transcripts were found in different SDT tissues. These differentially expressed transcripts were enriched in flavonoid and amino acid metabolism processes. Co-expression network analysis identified five modules associated with metabolites and found that genes of caffeine synthase (*TCS*) may be responsible for the low caffeine content in SDT. Phenylalanine ammonia lyase (*PAL*), glutamine synthetase (*GS*), glutamate synthase (*GOGAT*), and arginine decarboxylase (*ADC*) play important roles in the synthesis of catechin and theanine. In addition, we identified that ethylene resposive factor (*ERF*) and *WRKY* transcription factors may be involved in theanine biosynthesis. Overall, our study provides candidate genes to improve understanding of the synthesis mechanisms of these metabolites and provides a basis for molecular breeding of tea plant.

## Introduction

Tea plants are important economic crops that are rich in secondary metabolites. Their leaves can be made into six major tea types, such as green and black tea through different processing methods^1^. Owing to its good taste and health effects, tea is consumed worldwide^2,3^. Polyphenols, purine alkaloids, and theanine are the main characteristic metabolites of tea^4^. Catechins are the main polyphenols and include (+)-catechin (C), (−)-epicatechin (EC), (−)-epigallocatechin (EGC), (−)-epicatechin-3-gallate (ECG), and (−)-epigallocatechin-3-gallate (EGCG)^5^. Among these catechins, EGCG is the most important catechin component, accounting for more than 50% of total catechins in tea^6^. Caffeine (1,3,7-trimethylxanthine) is the most important purine alkaloid in tea and is usually used as one of the indicators for judging true and false tea^6^. However, due to its stimulating effect on the central nervous system, caffeine can cause insomnia and anxiety in individuals that are sensitive to such compounds^7^. Thus, there is a demand for caffeine-free or low-caffeine tea. At present, the caffeine synthesis pathway in tea plants is clear, and its direct synthesis gene, tea caffeine synthase (*TCS*), has also been cloned^8^. However, owing to the lack of a feasible genetic transformation system, it is difficult to breed caffeine-free tea varieties using biotechnological techniques. In addition, industrial decaffeination can reduce the biological activity and affect the taste of tea. Therefore, breeding high-quality low-caffeine tea varieties is of great significance.

Natural caffeine-free tea plant germplasms have been discovered previously, providing the possibility of using specific germplasms to breed caffeine-free tea varieties. Cocoa tea (*Camellia ptilophylla* Chang) is the first reported natural caffeine-free tea plant resource, which was found in the Guangdong Province of China and named due to its high accumulation of theobromine^9^. Subsequently, Jin et al.^10^ reported a new natural caffeine-free tea plant resource (‘Hongyacha’) in the Fujian Province. In our previous field investigation of tea plant resources, we established a new natural caffeine-free tea plant germplasm in the Sandu Aquatic Autonomous County of Guizhou (SDT) which was different from the above-mentioned reports. Specifically, based on the retrieval of the Section *Thea* classification system^11^, this tea variant is likely to belong to *Camellia costata* Chang. In previous research on tea plant resources in Guizhou province, the corresponding single nucleotide polymorphism (SNP) sites results from genotyping-by-sequencing (GBS) demonstrated that SDT was different from regular tea, and clustered with *Camellia costata*^12^. However, current knowledge of this resource is very limited. Considering the importance of specific resources for functional gene mining and their potential utilization in breeding, it is necessary to understand them more comprehensively.

Transcriptome sequencing is an effective method used to identify important functional genes^13^. Currently, next-generation and third-generation sequencing are widely used in transcriptome sequencing. Among them, next-generation sequencing is primarily based on PCR and gene chips, which have the advantages of high throughput and low cost^14^. Third-generation sequencing enables each DNA molecule to be sequenced separately in real-time with longer length of reads^15^. With the development of sequencing technology, single-molecular real-time (SMRT)-based full-length transcriptome sequencing technology has gradually been utilized in recent years. Compared with RNA-seq, it is easier to obtain full-length transcripts of genes in SMRT sequencing because of their long-read length characteristics; ultimately, this provides convenience when cloning full-length transcripts of genes and in the study of structural variation of transcripts^16^. An increasing number of genes related to growth and development, metabolite synthesis, and resistance have been identified in plants using second-generation sequencing, third-generation sequencing, or a combination of both^17–19^. Full-length transcriptome sequencing of peanuts has revealed its molecular regulatory mechanism under drought and salt stress^20^. In addition, combined analysis of transcriptome and metabolome data revealed the molecular mechanism for lignin accumulation in citrus fruits in response to oleocellosis-damage^21^. Sun et al.^22^ analyzed the transcriptome of pearl millet under heat and drought stress using next-generation sequencing combined with third-generation sequencing. There have also been many transcriptomic studies on secondary metabolic pathways and stress-related genes in tea plant^23,24^. For example, full-length transcripts and alternative splicing of secondary metabolics pathways in tea plants have been identified and analyzed through third-generation sequencing^25,26^. Many differentially expressed genes have been identified by RNA-seq of two tea cultivars with different cold resistance; consequently, under low temperature treatment the genes that responded to the key cold resistance pathways could be analyzed^27^. Additionally, alternative splicing genes in response to cold stress in tea plants have been identified and analyzed using the same sequencing data^28^. Therefore, the key differential genes of tea plant resources can be identified using transcriptome sequencing.

Here, we first identified the morphological characteristics of SDT by combining with the classification system of Section *Thea*. We identified the main secondary metabolites in different tissues of SDT by HPLC, and revealed its specificity by comparing this metabolite content with the content in the same leaf tissue of normal tea plants. The key genes of the main secondary metabolic pathways were further identified using full-length transcriptome sequencing and RNA-seq technology, and their expression patterns were compared with those in normal tea plants. Finally, a number of regulatory genes closely related to specific secondary metabolite content were discovered by weighted gene co-expression network analysis (WGCNA). These results deepened our understanding of the molecular basis of the specific traits of this novel, natural, caffeine-free resource, and established a foundation for the subsequent development and utilization of this tea plant.

## Materials and methods

### Plant materials

Tea plants were collected in the Sandu Aquatic Autonomous County of Guizhou (SDT) and preserved as tea germplasms in the Germplasm Tea Repository of the Guizhou Tea Research Institute located in Guiyang (N26°30′, E106°39′), Guizhou Province, China. Tea plants (*Camellia sinensis (L.) O. Kuntze cv. Qiancha 1*) cultivars Qiancha 1 (QC1) were grown at the Germplasm Tea Repository of the Guizhou Tea Research Institute. QC1 was used for comparison because it is considered to be a regular tea with normal caffeine content. One-year-old cuttings were sampled in the autumn (October) 2020. Five samples containing three biological replicates were collected. Tender leaf tissue samples from QC1 (QC1-TL) were collected and the corresponding SDT tissues samples were collected as follows: tender leaves (SDT-TL), mature leaves (SDT-ML), stem (SDT-S), root (SDT-R). Fresh tea samples were immediately frozen in liquid nitrogen and stored at − 80 °C.

### Determination of metabolite content

Catechins, alkaloids and theanine were extracted from all samples according to previously reported methods^29,30^. The extracts were filtered through a 0.22 μm membrane. Catechin and alkaloid were determined using an Agilent1100 HPLC system with a reverse phase C18 column (Phenomenex 250 mm × 4.6 mm, 5 μm). The extracts (10 μL) were injected into the HPLC system for analysis. The mobile phase was composed of A: DMF: methanol: acetic acid (40:2:1.5) and B: water at 1 mL/min; the effluent was monitored at 278 nm with column temperature of 35 ℃. The difference in theanine identification protocol was in flow phase A: SDS aqueous solution and acetonitrile, with detection at 203 nm. Samples from different tissues were analyzed in triplicate. All measured compounds were quantified using the standard curves of standard products.

### RNA extraction and sequencing

Total RNA was extracted from tea samples using RNeasy Plus Mini Kit (Tiangen), according to the manufacturer’s protocol. Then agarose gel electrophoresis and the Nanodrop 2500 (Thermo Fisher Scientific, US) were conducted to determine the quality and quantity of each RNA extract. High quality RNA extract was used for SMRT and next generation RNA sequencing.

The SMRT sequencing library was constructed using an equal mixture of RNAs from different SDT tissues. First-strand cDNA was synthesized using the SMARTer PCR cDNA Synthesis Kit and amplified by PCR. Then RNA-Seq libraries were constructed using a previously reported method^31^. After passing the library test, full-length transcriptome sequencing was performed by PacBio instrument (BioMarker, China). For next generation RNA-seq, mRNA from 15 tea samples was enriched by magnetic beads with Oligo (dT), then randomly interrupted; next, first-strand cDNA was synthesized, and then the library was constructed. Sequencing was conducted using Illumina the platform (BioMarker, China).

Raw SMRT sequencing data were analyzed using the SMRT analysis software package. The circular consensus sequence (CCS) was extracted according to full passes of ≥ 3 and sequence accuracy of > 0.9. Then, the CCS reads were divided into full-length and non-full-length sequences based on the presence of 5′primers, 3′primers, and a poly (A) tail. The IsoSeq module of SMRTLink software was used to cluster the similar sequences in the full-length non-chimeric sequences into a cluster. Finally, high quality transcripts with an accuracy > 99% were obtained. Proovread software was used to correct the low-quality consistent sequences based on Illumina RNA-seq data.

Clean reads were generated from next-generation sequencing data after removing adapter and primer sequences, and low-quality reads. High-quality full-length sequences and clean reads were used for subsequent analyses.

### Functional annotation of transcripts

The annotation information of transcripts was obtained by compared with the Non-redundant (Nr, http://www.ncbi.nlm.nih.gov/), Swissprot (http://ftp.ebi.ac.uk/pub/databases/swissprot), Clusters of Orthologous Groups (COG), Pfam (http://pfam.xfam.org), Gene Ontology (GO, http://geneontology.org), and Kyoto Encyclopedia of Genes and Genomes (KEGG, http://www.genome.jp/kegg) databases through Blast software (v.2.2.26). The Open Reading Frame (ORF) and corresponding amino acid sequences of the transcripts were predicted using TransDecoder software (https://github.com/TransDecoder/TransDecoder, v5.5.0). Only one coding sequence (CDS) per transcript was produced as an output by setting the “--single_best_only” parameter and homology search against the UniProt database.

### Identification of differentially expressed genes

The full-length transcripts obtained were used as a reference. Clean reads were mapped to the reference sequence using STAR software^32^ to obtain the location information of the transcripts. The fragments per kilobase of transcript per million fragments mapped (FPKM) were calculated using RSEM software^33^ and used to compare the expression levels of the transcripts.

Differential expression analysis was performed using the DEGseq R package^34^ with the FPKM value. Genes were considered to be differentially expressed genes (DEGs) if the p-value was < 0.01 and the fold-change was > 2. KEGG enrichment analysis of DEGs was performed using TBtools software based on KEGG annotations of full-length transcripts and plant KEGG background^35^. The name of the enrichment pathway, p-value, and gene number were visualized using the ggplot2 package^36^.

### Weighted gene co-expression network analysis

The data of 9752 DEGs and metabolite content were used to construct a weighted gene co-expression network analysis (WGCNA) using the the R WGCNA package^37^. An unsigned topological overlap matrix (TOM) was used to build the co-expression network. The detailed parameters are as follows: power = 24, minimum module size = 100, and branch merge cut height = 0.45. The edge weight and node connectivity of genes in several key modules were then calculated. The correlative relationships between genes in special modules were visualized using Cytoscape software^38^ (v.3.6.0).

### Ethical statement

All materials collected were not from endangered species and did not damage the local environment; all operations were conducted in accordance with the regulations of the relevant institution.

## Results

### Characteristics of morphological and chemical composition

**Figure 1:**
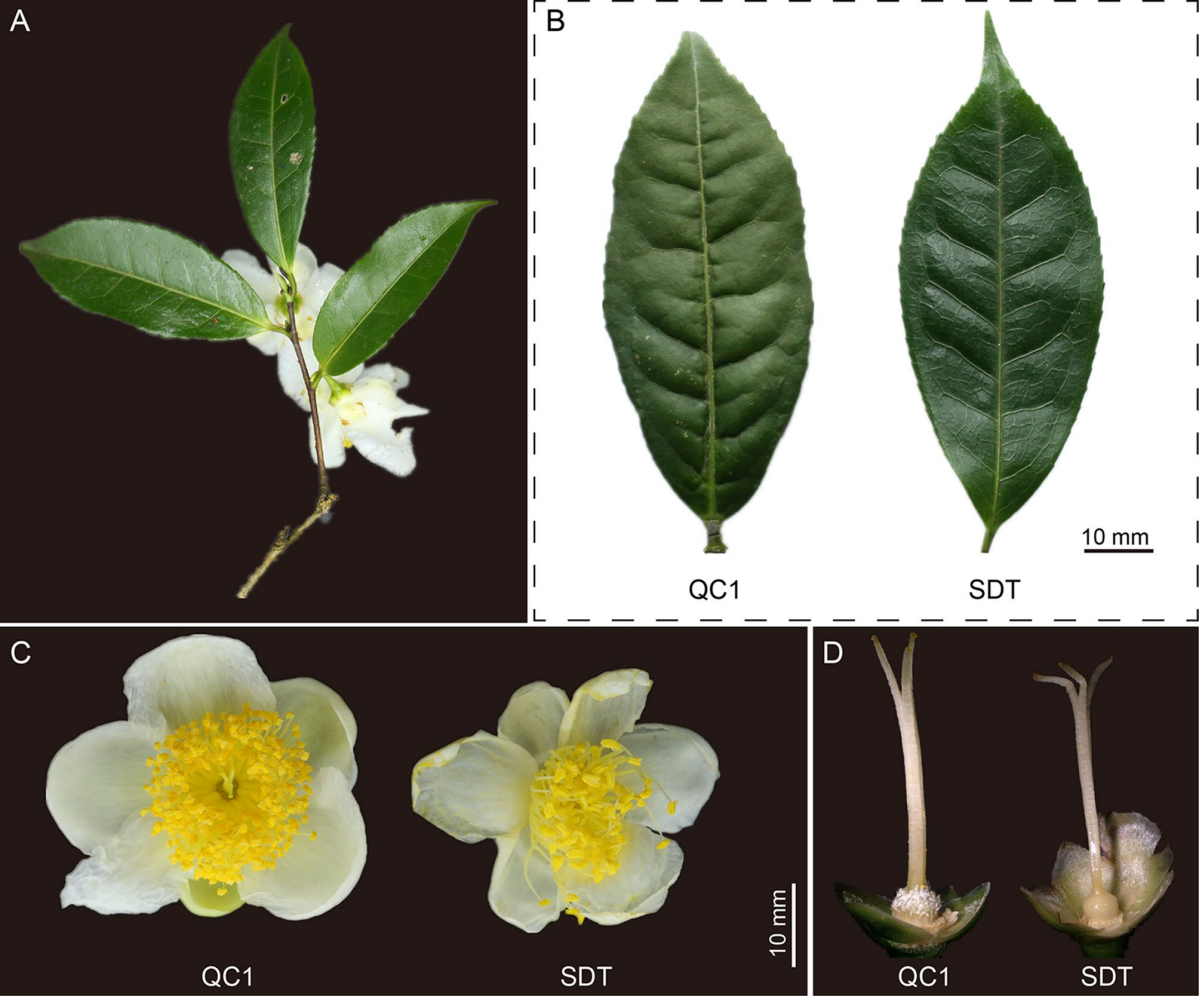
Morphological characteristics of ‘Qiancha 1’ and SDT. (**A**) SDT tea plant contains leaves, stem, and flowers. (**B**) Mature leaves of QC1 (left) and SDT (right). (**C**) Flower diameter of QC1 (left) and SDT (right). (**D**) Style and ovary of QC1 (left) and SDT (right). The image was taken by the author of this article without copyright problem.

**Figure 2:**
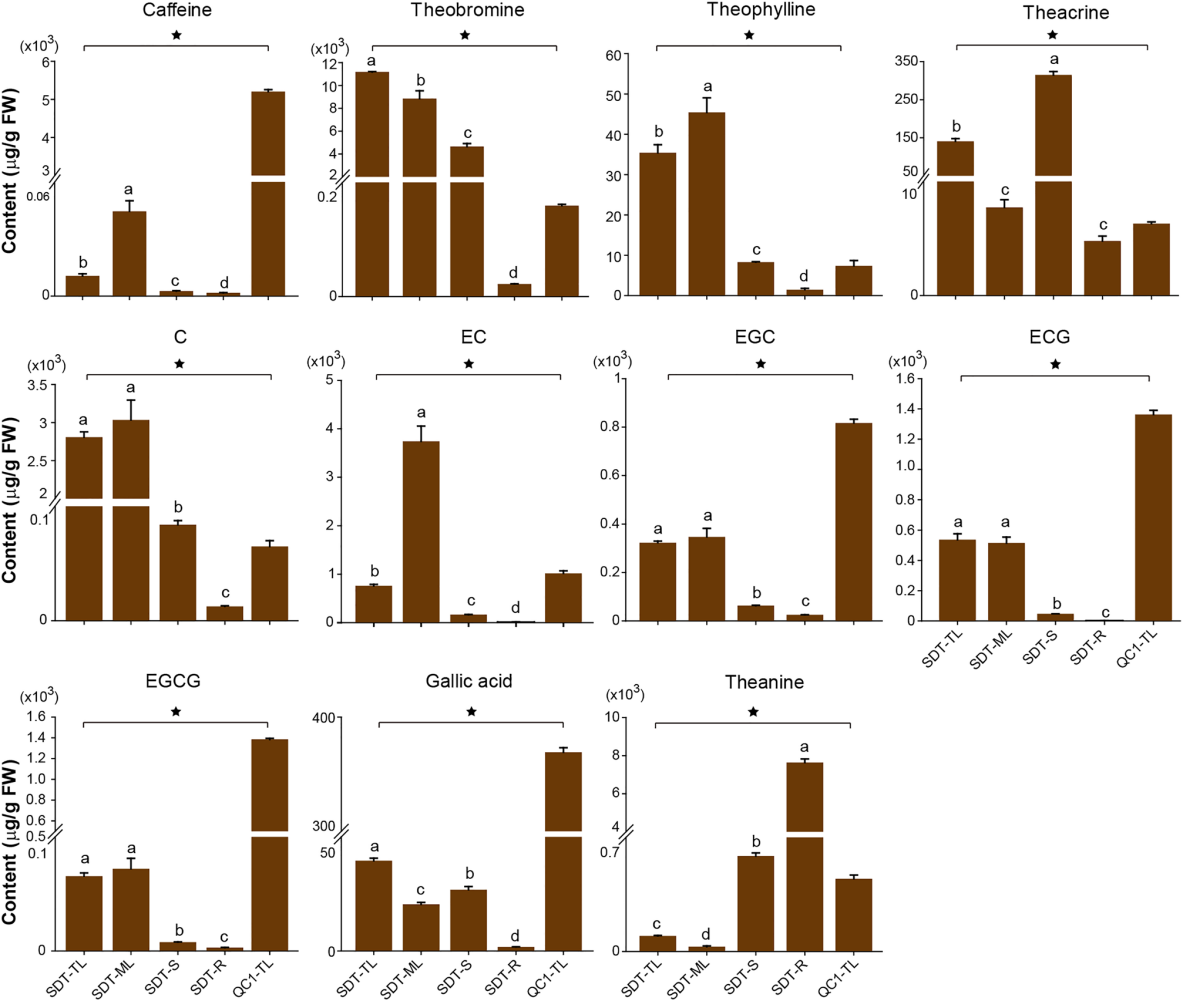
Contents of purine alkaloids, catechins and theanine in tea samples. Data represent mean ± SD of three biological replicates. The different lowercase letters on the bar graph indicate significant differences at p < 0.05.

The SDT tea variant belongs to the small arbor tree type. The mature leaves of SDT are green or yellow-green in color, narrow elliptic in shape, and leathery in texture (Fig. 1A; Supplementary Fig. S1A, B). The leaf base was wedge-shaped, and the leaf apex shape was acuminate (Fig. 1B; Supplementary Fig. S1C). The number of vein pairs was 7–9, and vein morphology was more prominent than that of QC1 (Fig. 1B). The pedicel was determined to be glabrous, and 6–8 mm in length (Supplementary Fig. S1D). The flower diameter of SDT was slightly smaller than that of QC1, varying from 2.0 to 3.5 cm. The number of petals ranged from 5 to 7 (Fig. 1C). The style and ovary of SDT were glabrous, and the style was trilobed at the apex (Fig. 1D; Supplementary Fig. S1F, G). The content of catechins, purine alkaloids, and theanine in different tissues of SDT was determined by HPLC. Additionally, the corresponding components in QC1 leaves at the same developmental stage were also detected to compare the difference between SDT and normal tea plants. The results demonstrated that the contents of these chemical components were significantly different between the two tea plants and different tissues of SDT. One of the most obvious features was that caffeine levels in SDT were extremely low. Specifically, the content of caffeine in QC1-TL was 5206.01 ± 49.4 μg/g, whereas a caffeine content of only 10.59 ± 1.05 μg/g was observed in SDT-TL; therefore, these QC1-TL caffeine levels were 500 times greater than that in SDT-TL (Fig. 2; Supplementary Fig. S2). The theobromine, theophylline, and theacrine content in SDT-TL were significantly higher than those in QC1-TL. Finally, the accumulation patterns of catechins in the two different tea plants tender leaves were divided into two types. The content of C in SDT-TL was higher than that in QC1-TL. In contrast, the levels of the ester catechins ECG and EGCG in QC1-TL were significantly higher than those in SDT-TL. The accumulation pattern of EGC was similar to that of the ester catechins in the tender leaves of QC1 and SDT plants. These metabolites are also different in different SDT tissues. Among the different tissues of SDT, caffeine was the highest in mature leaves (SDT-ML) at 44.53 ± 5.57 μg/g. Meanwhile, the content of the three purine alkaloids in the root (SDT-R) was the lowest than in all other SDT tissues. In addition, the content of all catechins in the leaves was significantly higher than that in the stems and roots (Fig. 2). In contrast, the highest content of theanine was 7638.83 ± 188.45 μg/g in SDT-R, followed by SDT-S, and SDT-TL, the lowest content was 29.66 ± 3.96 μg/g in SDT-ML.

### Assembly and functional annotation of the full-length transcriptome

To explore the relationship between metabolite differences and gene expression, we performed a transcriptome sequencing analysis of these samples. A library for full-length transcriptome sequencing was constructed by combining equal quantities of total RNA from all samples. PacBio sequencing generated 310,745 CCS reads after polishing, and the mean read length of the CCSs was 4219 bp. These CCS reads included 241,280 full-length non-chimeric read sequences. These sequences were then clustered, low-quality transcripts were filtered, and redundancy transcripts were removed to obtain 78,809 unigenes. To facilitate the subsequent analysis of these 78,809 transcripts, the unigenes were named transcript_1 to transcript_78809. BUSCO assessment revealed a total of 877 complete genes (60.9%), suggesting that the assembled transcriptome was relatively complete (Supplementary Fig. S3).

**Figure 3:**
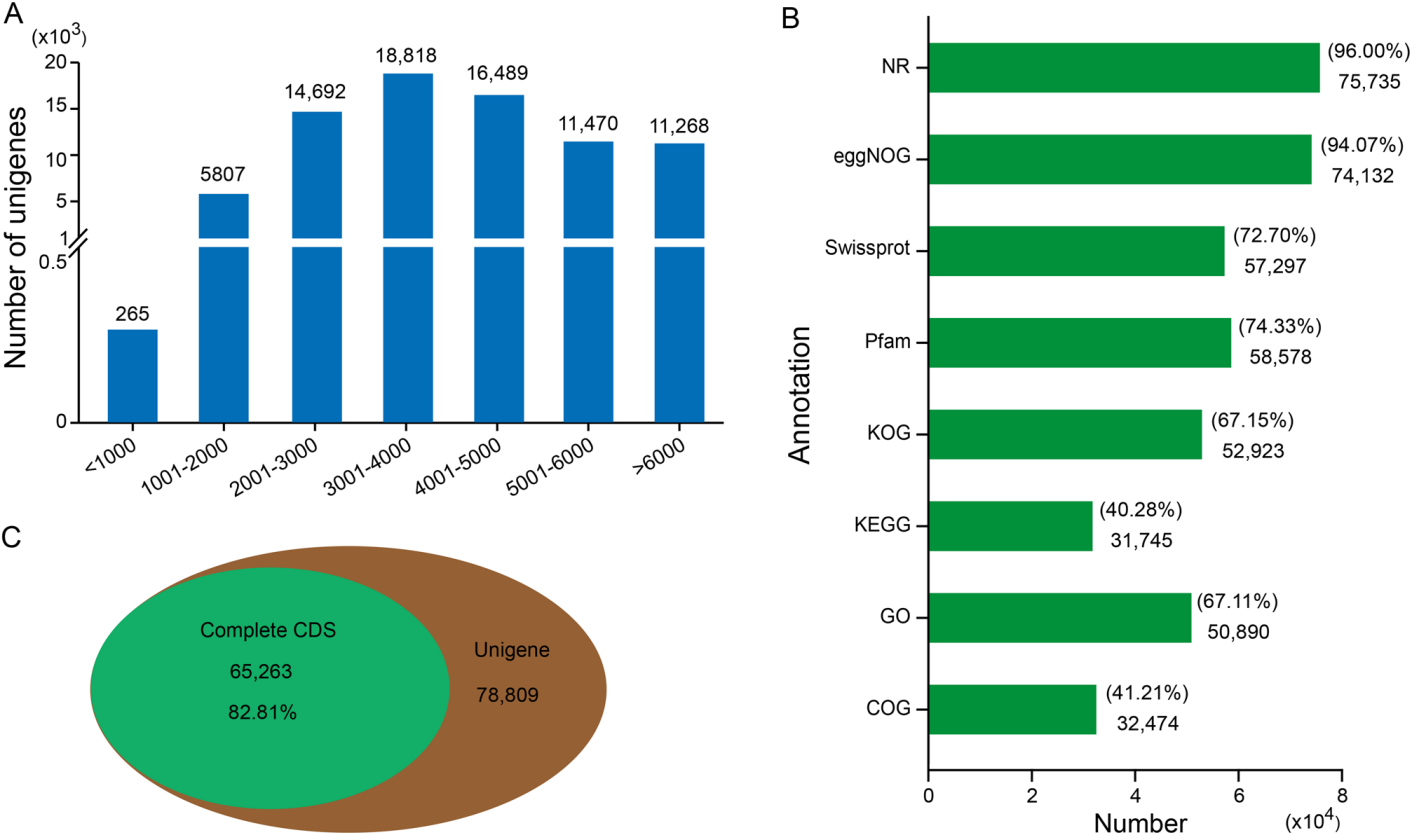
Characteristics of full-length transcriptome sequencing. (**A**) Length distribution of the unigenes. (**B**) Number and percentage of annotated genes in different database. (**C**) Number of complete coding sequences.

Overall, 85.7% (67,541) of these transcripts were found to be distributed within a length of 6 kb, among which a maximum of 18,818 were from 3001 to 4000 bp in length, while only 265 were within 1000 bp (Fig. 3A). To acquire annotation information for the transcripts, the sequences of the transcripts were aligned with the Nr, NOG, KEGG, COG, and GO databases. The results showed that 75,735, 74,132, 57,297, 58,578, 52,923, 31,745, 50,890, and 32,474 transcripts matched Nr, NOG, Swissport, Pfam, KOG, KEGG, GO, and COG databases, respectively. Furthermore, these transcripts accounted for 96.00%, 94.07%, 72.70%, 74.33%, 67.15%, 40.28%, 67.11%, and 41.21% of the total unigenes in the Nr, NOG, Swissport, Pfam, KOG, KEGG, GO and COG databases respectively (Fig. 3B). The longest ORF and amino acid sequences were predicted using TransDecoder software. In total, 65,263 complete CDS were identified, accounting for 82.81% of the total unigenes (Fig. 3C).

### Analysis of DEGs in different tissues

**Figure 4:**
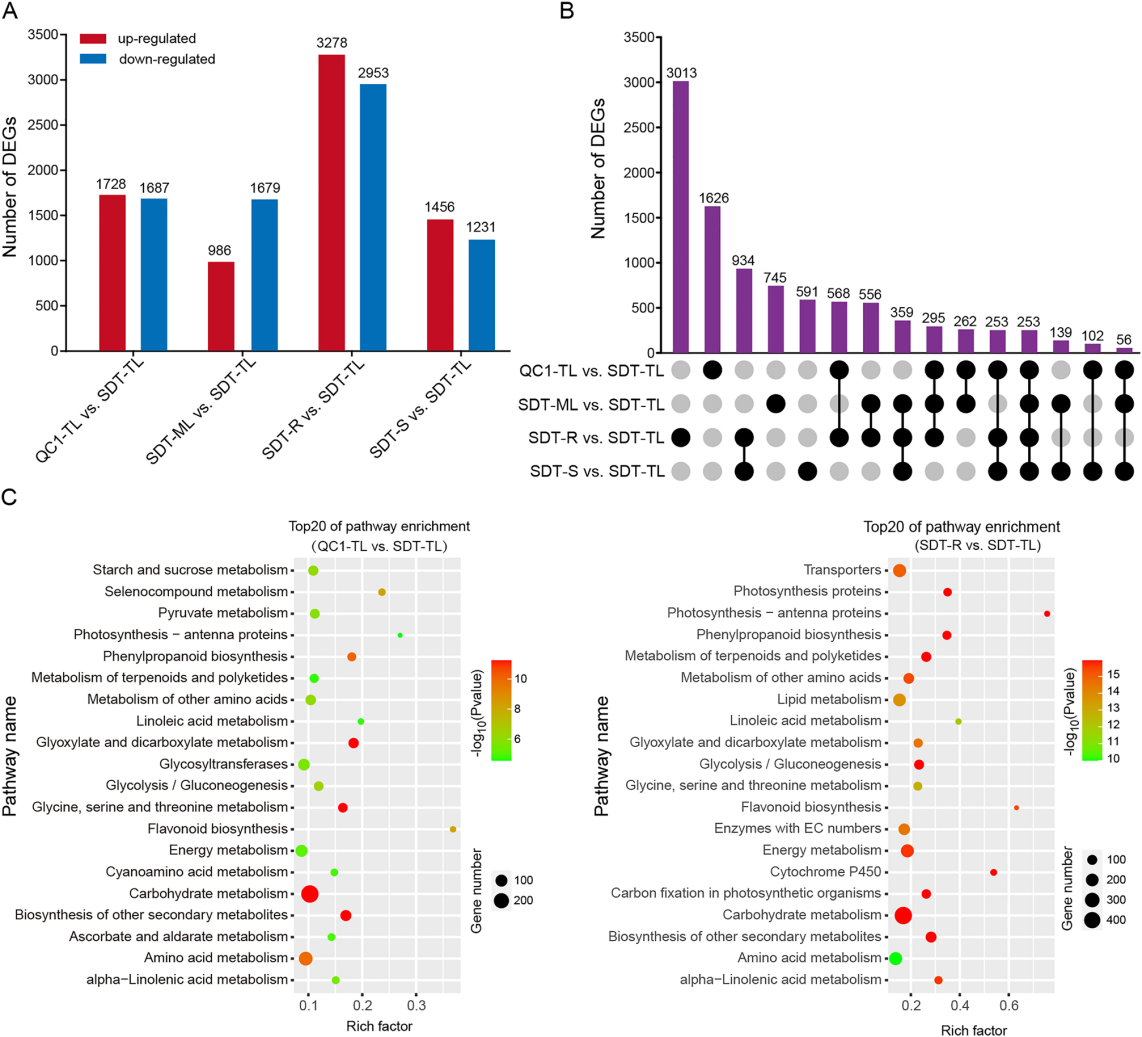
Analysis of differentially expressed genes (DEGs) by RNA-seq. (**A**) Statistics of DEGs in different samples. (**B**) Common and specific DEGs identified by upset venn diagram analysis. (**C**) KEGG enrichment analysis of DEGs in SDT-TL vs. QC1-TL and SDT-TL vs. SDT-R groups (p < 0.01). The size and color of the circles represent the number of DEGs and p-values, respectively. The x-axis represents the rich factor.

Fifteen tea plant samples were sequenced using the Illumina platform and were used to calculate the expression of unigenes. The reads obtained from these samples were between 5.8 and 7.6 Gb in size, the GC content was approximately 45%, and the Q30 quality score was > 90% (Supplementary Table S1). These findings indicated that the quantity and quality of sequencing were relatively high, which improved confidence in subsequent analyses. According to the standards discussed in the Methods, the DEGs in the tender leaves of QC1 and SDT, and different tissues in SDT were analyzed. Overall, 9752 DEGs were identified across all groups. The changes in these DEGs in each group were statistically significant (Fig. 4A). In total, 3415 DEGs were identified between QC1-TL and SDT-TL. We found that the number of DEGs in the SDT-R vs. SDT-TL group was the largest (6231 DEGs), among which 3278 were up-regulated and 2953 were down-regulated. In contrast, the number of DEGs in the SDT-ML vs. SDT-TL group was the least at 2665, among which 986 were up-regulated and 1679 were down-regulated. The number of up-regulated DEGs in the SDT-ML and SDT-TL groups was significantly smaller than that in down-regulated genes, whereas the other groups had more up-regulated genes (Fig. 4A). Moreover, the results demonstrated that most of the DEGs observed were specific to one group. For example, there were 3013,1626,745 and 591 unique genes in the SDT-R vs. SDT-TL, QC1-TL vs. SDT-TL, SDT-ML vs. SDT-TL and SDT-S vs. SDT-TL groups, respectively, while there were only 253 DEGs observed in all four groups (Fig. 4B). This indicated that a large number of DEGs were tissue-specific or variety-specific.

To further determine the functions of DEGs, KEGG enrichment analyses in the QC1-TL vs. SDT-TL and SDT-R vs. SDT-TL groups were performed. The top 20 enriched pathways were shown according to the p-value (p < 0.01) (Fig. 4C). DEGs were primarily enriched in the carbohydrate metabolism, secondary metabolites and amino acid metabolism in the QC1-TL vs. SDT-TL group. Transporters, Carbohydrate metabolism, flavonoid biosynthesis and amino acid pathway metabolism were enriched in the SDT-R vs. SDT-TL group. These results indicate that the DEGs affected the production of metabolites in the leaves of different tea plant and different tissues. To verify the sequencing results, nine genes were randomly selected for qRT-PCR analysis, the corresponding PCR results were consistent with the sequencing results (Supplementary Fig. S4).

### Analysis of genes associated with characteristic metabolite synthesis pathways

**Figure 5:**
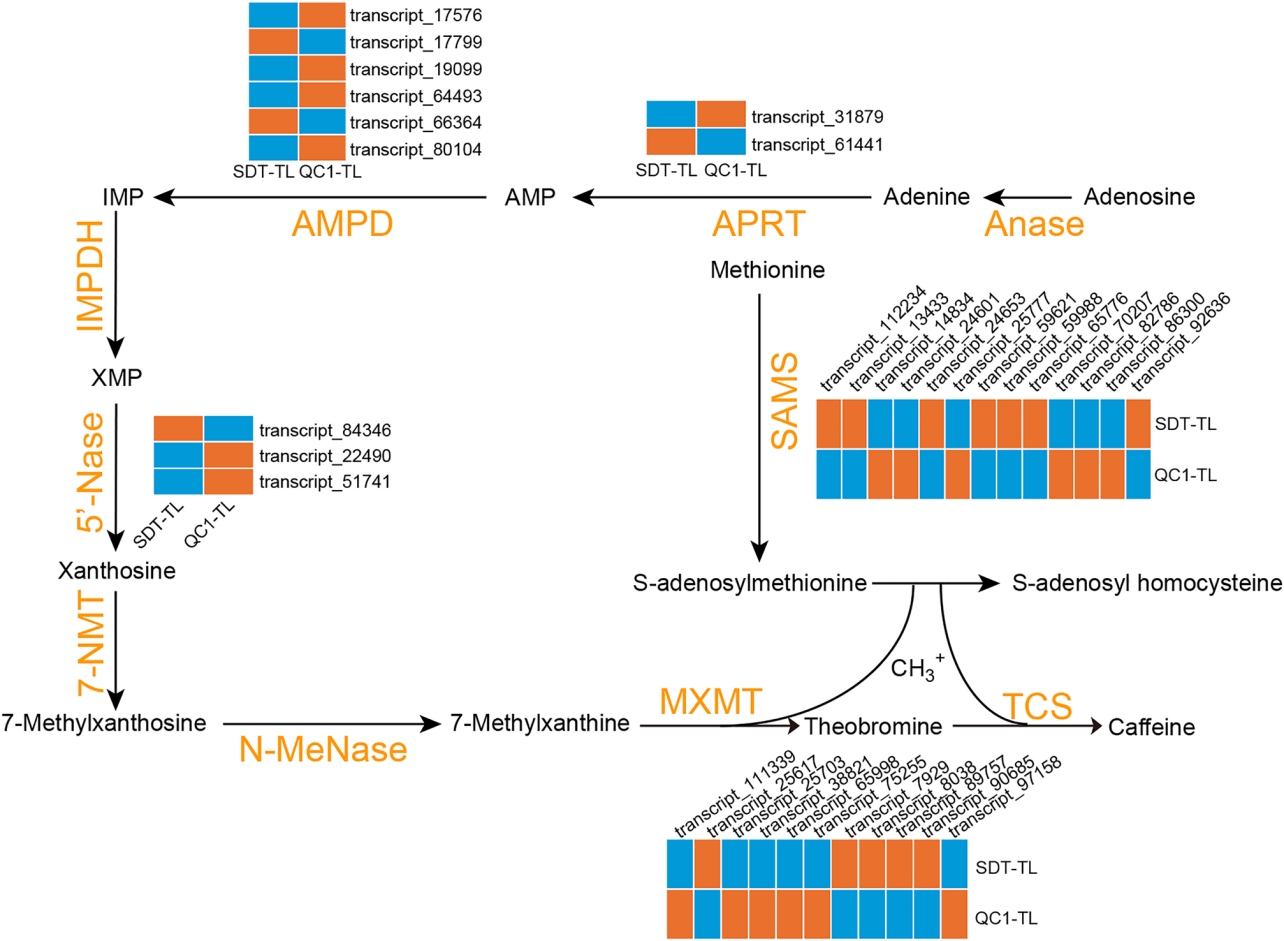
Biosynthetic pathway and gene expression analysis of caffeine metabolism. The orange letters indicate enzymes associated with the metabolites. Heat map showing the relative expression level of genes in QC1-TL and SDT-TL. Data represent the mean values of three biological replicates.

Through metabolite analysis, we established that SDT is a caffeine-free tea. To identify the key genes in SDT, we analyzed genes related to the biosynthetic pathways of its characteristic metabolites. The biosynthetic pathway of caffeine is part of purine metabolism and is initiated by the methyl donor S-adenosylmethionine (Fig. 5). Purine biosynthesis involves adenosine nucleosidase (Anase), adenine phosphoribosyl transferase (APRT), AMP deaminase (AMPDA), IMP dehydrogenase (IMPDH), and 5’ nucleotidase. Additionally, S-adenosylmethionine is synthesized by S-adenosine methionine synthase (SAMS). We identified one *APRT*, four *AMPD* and six *SAMS* genes that were highly expressed in QC1. (Fig. 5). The conversion of xanthosine to caffeine mainly involves three methylation steps. We identified 11 N-methyltransferases that differed between QC1 and SDT. Among them, six were highly expressed in QC1, which was consistent with the pattern of caffeine accumulation (Fig. 5). We then identified a sequence with the highest homology to the reported caffeine synthase gene and named it *TCS1* (transcript_65998). The corresponding TCS1 protein lacks five amino acids at the N-terminal, and is more similar to the reported caffeine synthase sequence in the caffeine-free resources of ‘hongyacha’ (HYC) and cocoa tea (CCT), with only five amino acids variations (Supplementary Fig. S5). In addition, genes related to the catechin and theanine synthesis pathways were analyzed (Supplementary Fig. S6). Three dihydroflavonol 4-reductase (*DFR*) and one leucoanthocyanidin reductase (*LAR*) genes were highly expressed in SDT, two serine carboxypeptidase-like acyltransferases (*SCPL*), two arginine decarboxylase (*ADC*) and eight glutamate synthase (*GOGAT*) genes were highly expressed in QC1.

### Co-expression analysis of differential metabolites and DEGs

**Figure 6:**
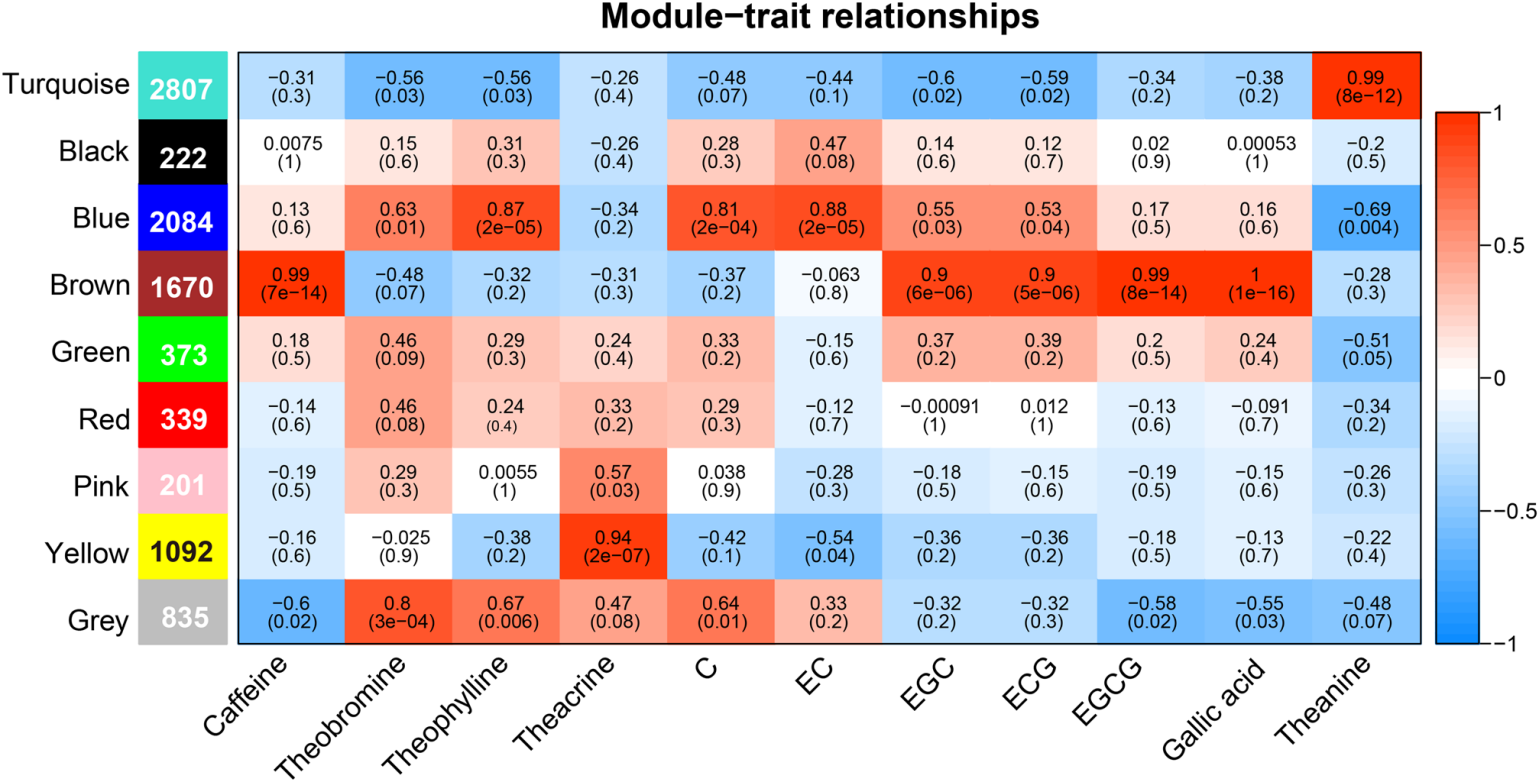
WGCNA analysis of DEGs. The left box indicates the number of genes in each module. The color and number of each cell indicate the correlation coefficient between the module and metabolite content, respectively. Blue and red colors indicate the negative and positive correlation between modules and compound content.

To explore the relationship between DEGs and the content of metabolites, a co-expression network was constructed using WGCNA. Nine modules were observed through clustering of the DEGs, and there was a strong correlation between the partial modules and metabolites (Fig. 6). Standard with r > 0.8 and p < 10^–3^, were set to identify key modules. Notably, genes in four modules (turquoise, blue, brown and yellow) were identified as key modules, and were significantly positively correlated with the content of metabolites (Fig. 6). For example, 2807 genes in the turquoise module were significantly positively correlated with theanine content (r = 0.99). A total of 2084 genes in the blue module were correlated with catechin and epicatechin. In addition, 1670 genes in the brown modules were significantly correlated with the content of theanine and ester catechin (ECG and EGCG) with r ≥ 0.9. Interestingly, all of these key modules were positively correlated with metabolite content, and no significantly negatively correlated gene modules were identified. This implies that the genes in these modules may participate directly in the synthesis of these metabolites.

**Figure 7:**
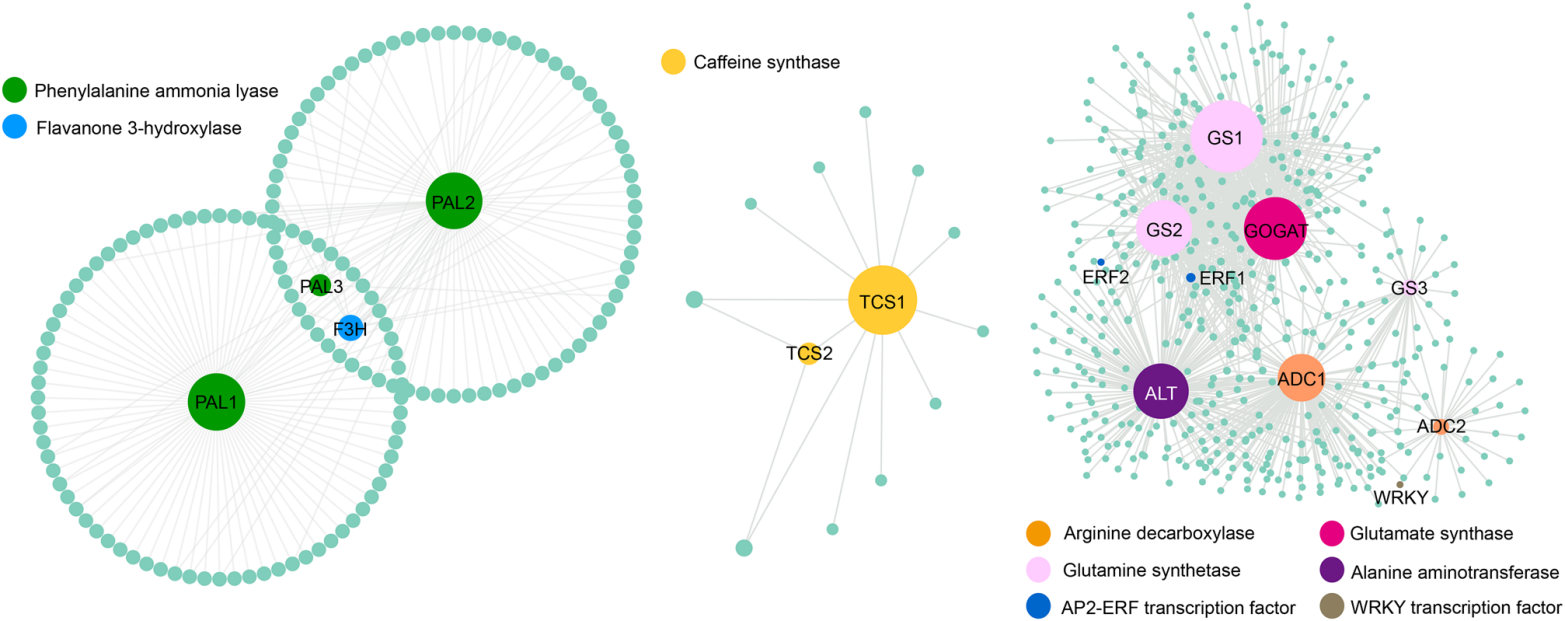
Co-expression network of DEGs related to taste compound biosynthesis in tea plants. The size and color of the circle represent the number of edges and different genes, respectively. This image was generated by Cytoscape software^38^ (v3.6.0, https://cytoscape.org/).

To construct the network, nodes associated with genes in the biosynthetic pathways of these compounds were selected from the WGCNA edge relationships. A total of 13 hub genes were selected from the turquoise, blue, and brown modules that were significantly associated with theanine, catechin, and theanine content (Fig. 7). The 13 hub genes, were mainly related to secondary metabolite synthesis pathways or transcription factors that may play a regulatory role in these pathways. In the blue module, three phenylalanine ammonia lyase (*PAL*) genes and one flavanone 3-hydroxylase (*F3H*) gene were significantly correlated with catechin and epicatechin content. In addition, two *TCS* genes related to caffeine content were identified in the brown module. Among them, *TCS1* was the differentially expressed gene identified in this study (transcript_65998). Through co-expression network analysis, we found that *GS*, *GOGAT*, *ADC* and alanine aminotransferase (*ALT*) genes were highly correlated with the content of theanine (Fig. 7; Supplementary Table S2). These genes are all related to glutamate and ethylamine, which are precursors of theanine synthesis. For example, we identified three *GS* and *GOGAT* genes that are associated with glutamic acid synthesis. Furthermore, through nodes analysis of these key genes, we identified four transcription factors that may play a potential regulatory role. Among them, ERF1 was strongly correlated with *GS1*, *GS2* and *GOGAT*, whereas *WRKY* was strongly correlated with *ADC2*.

## Discussion

Catechins, alkaloids and theanine are the characteristic compounds of tea. These compounds not only contribute to taste, but also possess various health effects^39,40^. Therefore, the synthesis and regulatory mechanisms of these compounds have been widely studied. In this study, we used a tea germplasm resource with low caffeine content to identify the key genes in these metabolic pathways through full-length transcriptome sequencing and RNA-seq. We constructed a co-expression network and found that *PAL*, *F3H*, *TCS*, *GS*, *GOGAT*, and *ADC* may play an important role in the synthesis of these compounds. Overall, this will provide a basis for the regulation of tea characteristic compounds genes and molecular breeding.

Caffeine is the main purine alkaloid, accounting for 2–3% of the dry weight of regular tea^41^. However, the caffeine content in SDT is almost undetectable. Therefore, SDT is a caffeine-free tea plant resource, similar to HYC and CCT. Compared with the two caffeine-free resources, the apex shape of mature leaves of SDT and HYC was acuminate. In addition, the styles of CCT and SDT are splitting, whereas the HYC style is almost not splitting. This shows that SDT is a novel caffeine-free resource with a divergent phenotype to other previously established caffeine-free variants. There are two main reasons for the low caffeine content. In a study of two caffeine-free tea plants, HYC and CCT, it was found that the amino acid sequence of their *TCS* was different from that of regular tea; therefore, these variants only had the biological activity of theobromine synthesis^10^. In addition to this class of theobromine rich caffeine-free resources, there is a natural non-caffeine tea plant resources that is rich in theacrine, and has a unique theacrine synthase (*TcS*) gene that can convert caffeine into theacrine; therefore no caffeine is observed in this tea plant variant^42^. In this study, we did not identify the *TcS* gene, but found a caffeine synthase (*TCS1*) gene that was significantly related to the corresponding low caffeine content in SDT. Through sequence alignment analysis, it was found that this gene had a conserved motif. Compared with the *TCS* of regular tea plants, *TCS1* lacked 5 amino acids at the N-terminal, which was consistent with HYC and CCT. Furthermore, the amino acid residues related to substrate recognition were consistent with these residues (Supplementary Fig. S5). These results suggest that *TCS1* in SDT may only exert biological activity affecting theobromine synthesis, but not in caffeine synthesis. Therefore, we speculated that the reason for the low caffeine content in SDT may be similar to that of ‘hongyacha’ and cocoa tea. Second, the expression level of *TCS1* in SDT was also relatively low. Therefore, the expression pattern and biological activity of *TCS1* in SDT may explain the low caffeine content in SDT. Caffeine is the predominant purine alkaloid in regular tea, while theobromine content is relatively low^43^. In contrast, theobromine was the most dominant purine alkaloid in the SDT. As a typical non-caffeinated tea, the main purine alkaloid in cocoa tea is theobromine, with theophylline as a minor component^44^. Consistent with cocoa tea, the content of theobromine in SDT was the highest, and theophylline was also rich. Furthermore, a relatively high content of bitter theobromine was detected in SDT. In regular tea, the main catechin is EGCG^45^, but the contents of EGCG, EGC, and ECG in SDT are relatively low, whereas the content of C is relatively high. Cocoa tea also has higher levels of C, but lower levels of EGCG, and mainly contains GCG^46^. Theanine is a unique amino acid found in teas. We detected theanine in SDT, which accumulated in the roots, but the content in the leaves was significantly lower than that in regular tea. Because of its unique metabolite characteristics, the extract of cocoa tea has anti-obesity and anti-cancer effects^47,48^. The characteristic metabolite profile of SDT was similar to that of cocoa tea, thus, SDT tea may be a potentially beneficial drink for health.

Phenylalanine ammonia lyase is the key rate-limiting enzyme in phenylpropane metabolism and is the link between primary and secondary metabolism^49^. Many studies have confirmed that *PAL* is involved in theanine biosynthesis^50^. In this study, three *PAL* genes were identified that may be involved in the regulation of catechin biosynthesis. Glutamic acid and ethylamine are considered the precursors of theanine synthesis. Studies have shown that ethylamine content is highly positively correlated with theanine, and *CsAlaDC* and *CsGDH2.1* genes related to theanine synthesis precursors can regulate theanine biosynthesis^51,52^. We identified a large number of genes related to theanine synthesis, such as *ADC*, *GOGAT*, and *ALT*. These genes may also affect theanine content by regulating the biosynthesis of glutamic acid and ethylamine. In addition, we found *ERF* and *WRKY* transcription factor genes at the nodes of these hub genes. This suggests that *ERF* and *WRKY* transcription factors may affect theanine synthesis by regulating the expression of these genes.

## Supplementary Information

Supplementary Figure S1.

Supplementary Figure S2.

Supplementary Figure S3.

Supplementary Figure S4.

Supplementary Figure S5.

Supplementary Figure S6.

Supplementary Table S1.

Supplementary Table S2.

## Data Availability

The original data generated by sequencing presented in this study have been deposited at https://ngdc.cncb.ac.cn/gsa (GSA: CRA008658).

## References

[1] Xia E (2020). The reference genome of tea plant and resequencing of 81 diverse accessions provide insights into its genome evolution and adaptation. Mol. Plant.

[2] Kamal DAM, Salamt N, Zaid SSM, Mokhtar MH (2021). Beneficial effects of green tea catechins on female reproductive disorders: A review. Molecules.

[3] Qiao D (2021). Integrated metabolic phenotypes and gene expression profiles revealed the effect of spreading on aroma volatiles formation in postharvest leaves of green tea. Food Res. Int..

[4] Gu C, Wang R, Jiang L, Deng W (2017). Short-term shading influencing the biosynthesis of caffeine, theanine and catechins in tea (*Camellia sinensis*). J. Anhui Agric. Univ..

[5] de-Lourdes-Mata-Bilbao M (2007). A new LC/MS/MS rapid and sensitive method for the determination of green tea catechins and their metabolites in biological samples. J. Agric. Food Chem..

[6] Ning J (2016). Stepwise identification of six tea (*Camellia sinensis* (L.)) categories based on catechins, caffeine, and theanine contents combined with fisher discriminant analysis. Food Anal. Methods.

[7] Smith A (2002). Effects of caffeine on human behavior. Food Chem. Toxicol..

[8] Kato M, Mizuno K, Crozier A, Fujimura T, Ashihara H (2000). Caffeine synthase gene from tea leaves. Nature.

[9] Lin X (2014). Interactions among chemical components of Cocoa tea (*Camellia ptilophylla* Chang), a naturally low caffeine-containing tea species. Food Funct..

[10] Jin JQ (2018). Hongyacha, a naturally caffeine-free tea plant from Fujian, China. J. Agric. Food Chem..

[11] Chang, H. T. Thea—a section of beveragial tea-trees of the genus Camellia. In *Acta Scientiarum Naturalium Universitatis Sunyatseni* (1981).

[12] Guo C (2021). Genome-wide SNP developed by genotyping-by-sequencing revealed the phylogenetic relationship of Sect Thea(L.) Dyer resources in Guizhou. J. Southern Agric..

[13] Song F (2022). Transcriptome and association mapping revealed functional genes respond to drought stress in Populus. Front. Plant Sci..

[14] Forde BM, O’Toole P (2013). Next-generation sequencing technologies and their impact on microbial genomics. Brief. Funct. Genom..

[15] Jayakumar V, Sakakibara Y (2019). Comprehensive evaluation of non-hybrid genome assembly tools for third-generation PacBio long-read sequence data. Brief. Bioinform..

[16] Anthony R, Fai AK (2019). PacBio sequencing and its applications. Genom. Proteom. Bioinform..

[17] Li S (2018). Modulating plant growth–metabolism coordination for sustainable agriculture. Nature.

[18] Li S (2021). Metabolic and transcriptomic analyses reveal different metabolite biosynthesis profiles between leaf buds and mature leaves in *Ziziphus jujuba* mill. Food Chem..

[19] Schaarschmidt S (2020). Utilizing PacBio iso-seq for novel transcript and gene discovery of abiotic stress responses in *Oryza sativa* L.. Int. J. Mol. Sci..

[20] Zhao C (2021). De novo full length transcriptome analysis of *Arachis glabrata* provides insights into gene expression dynamics in response to biotic and abiotic stresses. Genomics.

[21] Zhou X (2021). Integration of metabolome, histochemistry and transcriptome analysis provides insights into lignin accumulation in oleocellosis-damaged flavedo of citrus fruit. Postharvest. Biol. Technol..

[22] Sun M (2020). Transcriptome analysis of heat stress and drought stress in pearl millet based on Pacbio full-length transcriptome sequencing. BMC Plant Biol..

[23] Fz B (2021). Transcriptome analysis identifies CsNRT genes involved in nitrogen uptake in tea plants, with a major role of CsNRT24. Plant Physiol. Biochem..

[24] Wang J (2019). The transcriptome analysis of different tea cultivars in response to the spring cold spells. J. Tea Sci..

[25] Dahe G (2019). Comprehensive identification of the full-length transcripts and alternative splicing related to the secondary metabolism pathways in the tea plant (*Camellia sinensis*). Sci. Rep..

[26] Xu Q (2017). *Camellia sinensis* transcriptome profiling using single-molecule direct RNA sequencing approach for in-depth understanding of genes in secondary metabolism pathways of. Front. Plant Sci..

[27] Li Y (2019). Comparative transcriptomic analysis reveals gene expression associated with cold adaptation in the tea plant *Camellia sinensis*. BMC Genom..

[28] Li Y (2020). Comprehensive profiling of alternative splicing landscape during cold acclimation in tea plant. BMC Genom..

[29] Zhao S (2020). The biosynthesis of main taste compounds is coordinately regulated by miRNAs and phytohormones in tea plant (*Camellia sinensis*). J. Agric. Food Chem..

[30] Shan Y (2011). Catechins synthesis and accumulation in tea seedlings at different development stages. J. Anhui Agric. Univ..

[31] Schaarschmidt S (2020). Utilizing PacBio iso-seq for novel transcript and gene discovery of abiotic stress responses in *Oryza sativa* L.. Int. J. Mol. Sci..

[32] Dobin A (2013). STAR: Ultrafast universal RNA-seq aligner. Bioinformatics.

[33] Li B, Dewey CN (2011). RSEM: Accurate transcript quantification from RNA-Seq data with or without a reference genome. BMC Bioinform..

[34] Wang L, Feng Z, Wang X, Wang X, Zhang X (2010). DEGseq: An R package for identifying differentially expressed genes from RNA-seq data. Bioinformatics.

[35] Kanehisa M, Goto S, Furumichi M, Tanabe M, Hirakawa M (2010). KEGG for representation and analysis of molecular networks involving diseases and drugs. Nucleic Acids Res..

[36] Wickham, H. *Ggplot2: Elegant Graphics for Data Analysis* (ggplot2: Elegant Graphics for Data Analysis, 2009).

[37] Langfelder P, Horvath S (2008). WGCNA: An R package for weighted correlation network analysis. BMC Bioinform..

[38] Shannon P (2003). Cytoscape: A software environment for integrated models of biomolecular interaction networks. Genome Res..

[39] Zhang L, Cao QQ, Granato D, Xu YQ, Ho CT (2020). Association between chemistry and taste of tea: A review. Trends Food Sci. Technol..

[40] Vuong QV (2014). Epidemiological evidence linking tea consumption to human health: A review. Crit. Rev. Food Sci. Nutr..

[41] Tadahiro N, Shinsuke S (1984). Differences in caffeine, flavanols and amino acids contents in leaves of cultivated species of camellia. Jpn. J. Breed..

[42] Zhang Y-H (2020). Identification and characterization of N9-methyltransferase involved in converting caffeine into non-stimulatory theacrine in tea. Nat. Commun..

[43] Wang D, Lu J, Miao A, Xie Z, Yang D (2008). HPLC-DAD-ESI-MS/MS analysis of polyphenols and purine alkaloids in leaves of 22 tea cultivars in China. J. Food Compos. Anal..

[44] Ashihara H, Kato M, Chuang-xing Y (1998). Biosynthesis and metabolism of purine alkaloids in leaves of cocoa tea (*Camellia ptilophylla*). J. Plant. Res..

[45] Jin J-Q, Ma J-Q, Ma C-L, Yao M-Z, Chen L (2014). Determination of catechin content in representative Chinese tea germplasms. J. Agric. Food Chem..

[46] Peng L (2011). Characterization of the constituents and antioxidative activity of cocoa tea (*Camellia ptilophylla*). Food Chem..

[47] Li P, Khan N, Afaq F, Mukhtar YH (2010). In vitro and in vivo effects of water extract of white cocoa tea (*Camellia ptilophylla*) against human prostate cancer. Pharmaceut. Res..

[48] Yang XR (2013). Effect of dietary cocoa tea (*Camellia ptilophylla*) supplementation on high-fat diet-induced obesity, hepatic steatosis, and hyperlipidemia in mice. Evid. Based Complement. Altern. Med..

[49] Zhang LW (2009). Molecular cloning and sequence analysis of mulberry phenylalanine ammonia-lyase gene. Sci. Sericult..

[50] Liu M (2015). Relationship between gene expression and the accumulation of catechin during spring and autumn in tea plants (*Camellia sinensis* L.). Horticult. Res..

[51] Zhu B (2021). CsAlaDC and CsTSI work coordinately to determine theanine biosynthesis in tea plants (*Camellia sinensis* L.) and confer high levels of theanine accumulation in a non-tea plant. Plant Biotechnol. J..

[52] Chen T (2022). CsGDH2.1 negatively regulates theanine accumulation in the late-spring tea plants (*Camellia sinensis* var. sinensis). Horticult. Res..

