# Supplementary material for: De novo full length transcriptome analysis of a naturally caffeine-free tea plant reveals specificity in secondary metabolic regulation
Source: Sci Rep. 2023 Apr 12;13:6015. doi: 10.1038/s41598-023-32435-5 (PMC10097665; doi:10.1038/s41598-023-32435-5)
Supplement: Supplementary file 1 — Supplementary Figure S1. [file 41598_2023_32435_MOESM1_ESM.pdf]

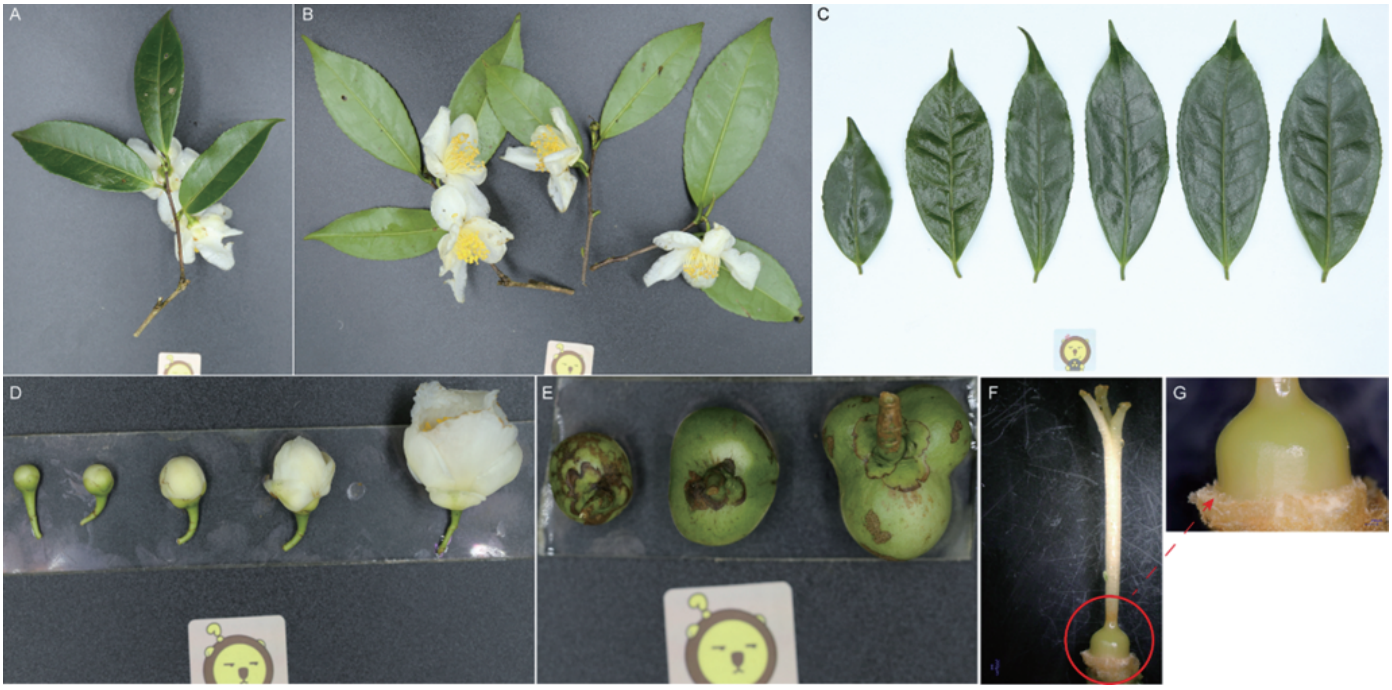

Figure S1. Morphological characteristics of ‘SDT’. (A) Branchlet and leaves surface. (B) Branch and blade back. (C) Morphology of leaves. (D) Pedicel and flowers at different developmental stages. (E) Fruits. (F) Style and ovary. (G) Ovary.
