# Supplementary material for: De novo full length transcriptome analysis of a naturally caffeine-free tea plant reveals specificity in secondary metabolic regulation
Source: Sci Rep. 2023 Apr 12;13:6015. doi: 10.1038/s41598-023-32435-5 (PMC10097665; doi:10.1038/s41598-023-32435-5)
Supplement: Supplementary file 2 — Supplementary Figure S2. [file 41598_2023_32435_MOESM2_ESM.pdf]

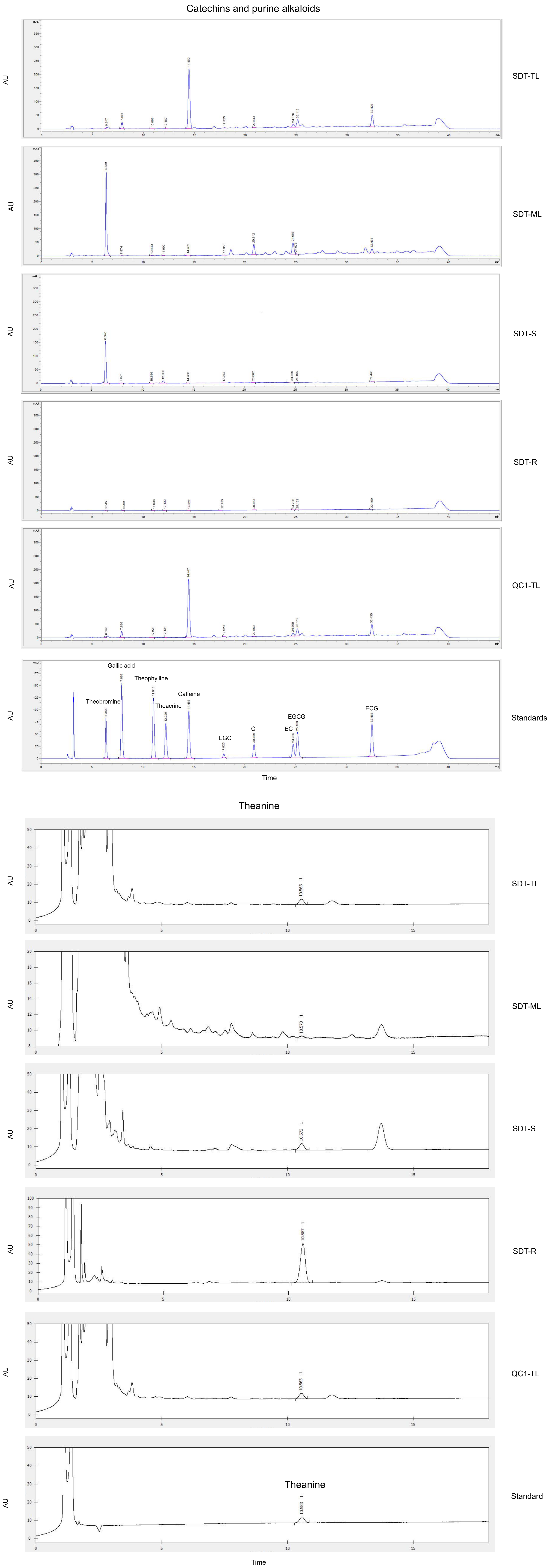

Figure S2. HPLC chromatogram of catechins, purine alkaloids and theanine in QC1 and SDT.
