# Supplementary figures and images for: De novo full length transcriptome analysis of a naturally caffeine-free tea plant reveals specificity in secondary metabolic regulation
Source: Sci Rep. 2023 Apr 12;13:6015. doi: 10.1038/s41598-023-32435-5 (PMC10097665; doi:10.1038/s41598-023-32435-5)

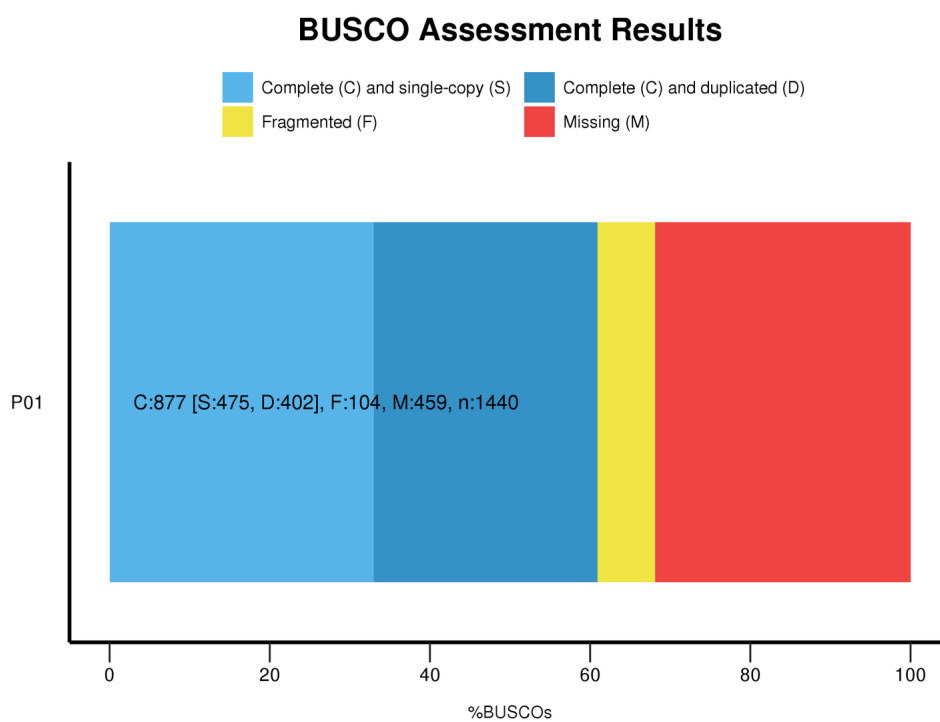

Figure S3. BUSCO assessment of assembled full-length transcriptome.

Supplement: Supplementary file 3 — Supplementary Figure S3. [file 41598_2023_32435_MOESM3_ESM.pdf]

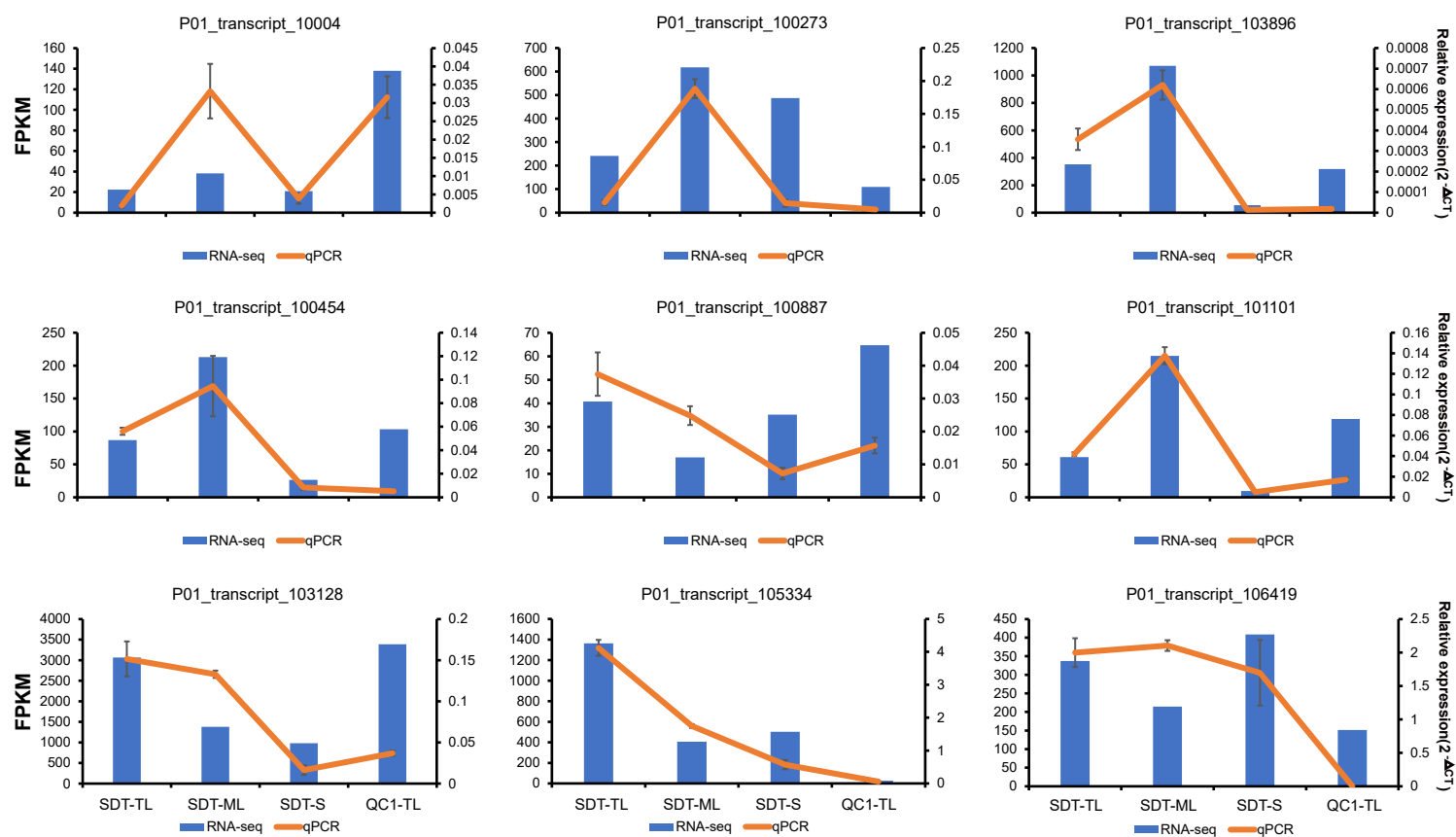

Figure S4. Analysis of DEGs by RNA-seq and qRT-PCR.

Supplement: Supplementary file 4 — Supplementary Figure S4. [file 41598_2023_32435_MOESM4_ESM.pdf]
