# Supplementary material for: De novo full length transcriptome analysis of a naturally caffeine-free tea plant reveals specificity in secondary metabolic regulation
Source: Sci Rep. 2023 Apr 12;13:6015. doi: 10.1038/s41598-023-32435-5 (PMC10097665; doi:10.1038/s41598-023-32435-5)
Supplement: Supplementary file 5 — Supplementary Figure S5. [file 41598_2023_32435_MOESM5_ESM.pdf]

```
TCS1a    MELATAGKVNEVLFMNRGEGESS.YAQNSSFTQQVASMAQPALENAVETLFSRDFHLQAL    59
HYC      .....MGKVNEVLFMNRGEGEITSYAQNSAFTQKVASMAMPALENAVETLFSKDFHLLQA    55
CCT      .....MGKVNEVLFMNRGEGEITSYAQNSAFTQKVASMAMPALENAVETLFSKDFHLLQA    55
TCS1     .....MGKVNEVLFMNRGEGEIS.YAQNSAFTQKVASMAMPALENAVETLFSKDFHLLQA    54
Consensus     gkvnevlfmnrgege   yaqns ftq vasma palenavetlfs dfhl

TCS1a    N.AADLGCAAGPNTFAVISTIKRMMEKKCRELNCQTLELQVYLNDLFGNDFNTLFKGLSS    118
HYC      LNAADLGCAAGPNTFAVISTIKRMMEKKCRELYCQTLELQVYLNDLFGNDFNTLFKGLSS    115
CCT      LNAADLGCAAGPNTFAVISTIKRMMEKKCRELYCQTLELQVYLNDLFGNDFNTLFKGLSS    115
SDT      LTAADLGCAAGPNTFAVISTIKRMMEKKCRELYCQTLELQVYLNDLFGNDFNTLFKGLSS    114
Consensus  aadlgcaagpntfavistikrmmekkcrel cqtlelqvylndlfgndfntlfkglss

TCS1a    EVIGNKCEEVPCYVMGVPGSFHGRLFPRNSLHLVHSSYSVHWLTQAPKGLTSREGLALNK    178
HYC      QVVGNKCEEVSCYVMGVPGSFHGRLFPRNSLHLVHSSYSVHWLTQAPKGLTSREGLALNK    175
CCT      QVVGNKCEEVSCYVMGVPGSFHGRLFPRNSLHLVHSSYSVHWLTQAPKGLTSREGLALNK    175
SDT      EVVGNKCEEVSCYVMGVPGSFHGRLFPRNSLHLVHSSYSVHWLTQAPKGLTSREGLALNK    174
Consensusv gnkceev cyvmgvpgsfhgrlfprnslhlvhssysvhwltqapkgltsreglalnk

TCS1a    GKIYISKTSPPVVREAYLSQFHEDFTMFLNARSQEVVPNGCMVLILRGRQCSDPSDMQSC    238
HYC      GKIYISKTSPPVVKEAYLSQFHEDFTMFLNARSQEVVPNGCMVLILHGRQSSDPSEMESC    235
CCT      GKIYISKTSPPVVKEAYLSQFHEDFTMFLNARSQEVVPNGCMVLILHGRQSSDPSEMESC    235
SDT      GKIYISKTSPPVVKEAYLSQFHEDFTMFLNARSQEVVPNGCMVLILHGRQSSDPSEMESC    234
Consensusugkiyisktsppvv eaylsqfhedftmflnarsqevvpngcmvlil grq sdps m sc

TCS1a    FTWELLAMAIAELVSQGLIDEDKLDTFNIPSYFASLEEVKDIVERDGSFTIDHIEGFDLD    298
HYC      FTWELLAIAIAELVSQGLIDEDKLDTFNVPSYWPSLEEVKDIVERDGSFTIDRLEGFELD    295
CCT      FTWELLAIAIAELVSQGLIDEDKLDTFNVPSYWPSLKEVKDIVERDGSFTIDHLEGFELD    295
SDT      STWELLAMAIAELVSQGLIDEDKLDTFNVPSYWPSLEEVKDIVERDGSFTIDHLEGFELD    294
Consensusustwella aiaelvsqglidedkldtfn psy  sl evkdiverdgsftid  egf ld

TCS1a    SVEMQENDKWVRGEKFTKVVRAFTEPIISNQFGPEIMDKLYDKFTHIVVSDLEAKLPKTT    358
HYC      SLEMQENDKWVRGDKFAKMVRAFTEPIISNQFGHEIMDKLYDKFTHIVLSDLEAELPKTT    355
CCT      SLEMQENDKWVRGDKFAKMVRAFTEPIISNQFGHEIMDKLYDKFTHIVLSDLEAELPKTT    355
SDT      SLEMQENDKWVRGDKFAKMVRAFTEPIISNQFGHEIMDKLYDKFTHILVSDLGAELPKTT    354
Consensus emqendkwvrg kf k vraftepiisnqfg eimdklydkfthi  sdl a lpktt

TCS1a    SIILVLSKID                                                      368
HYC      SIILVLSKIV                                                      365
CCT      SIILVLSKIV                                                      365
SDT      SIILVLSKIV                                                      364
Consensusiilvlski
```

Figure S5. Amino acid sequence analysis of caffeine synthase gene.The red box represents consered motif. Green circle has a critical role in substrate recognition. Red circle represents different amino acid sequences.
