# Supplementary material for: De novo full length transcriptome analysis of a naturally caffeine-free tea plant reveals specificity in secondary metabolic regulation
Source: Sci Rep. 2023 Apr 12;13:6015. doi: 10.1038/s41598-023-32435-5 (PMC10097665; doi:10.1038/s41598-023-32435-5)
Supplement: Supplementary file 6 — Supplementary Figure S6. [file 41598_2023_32435_MOESM6_ESM.pdf]

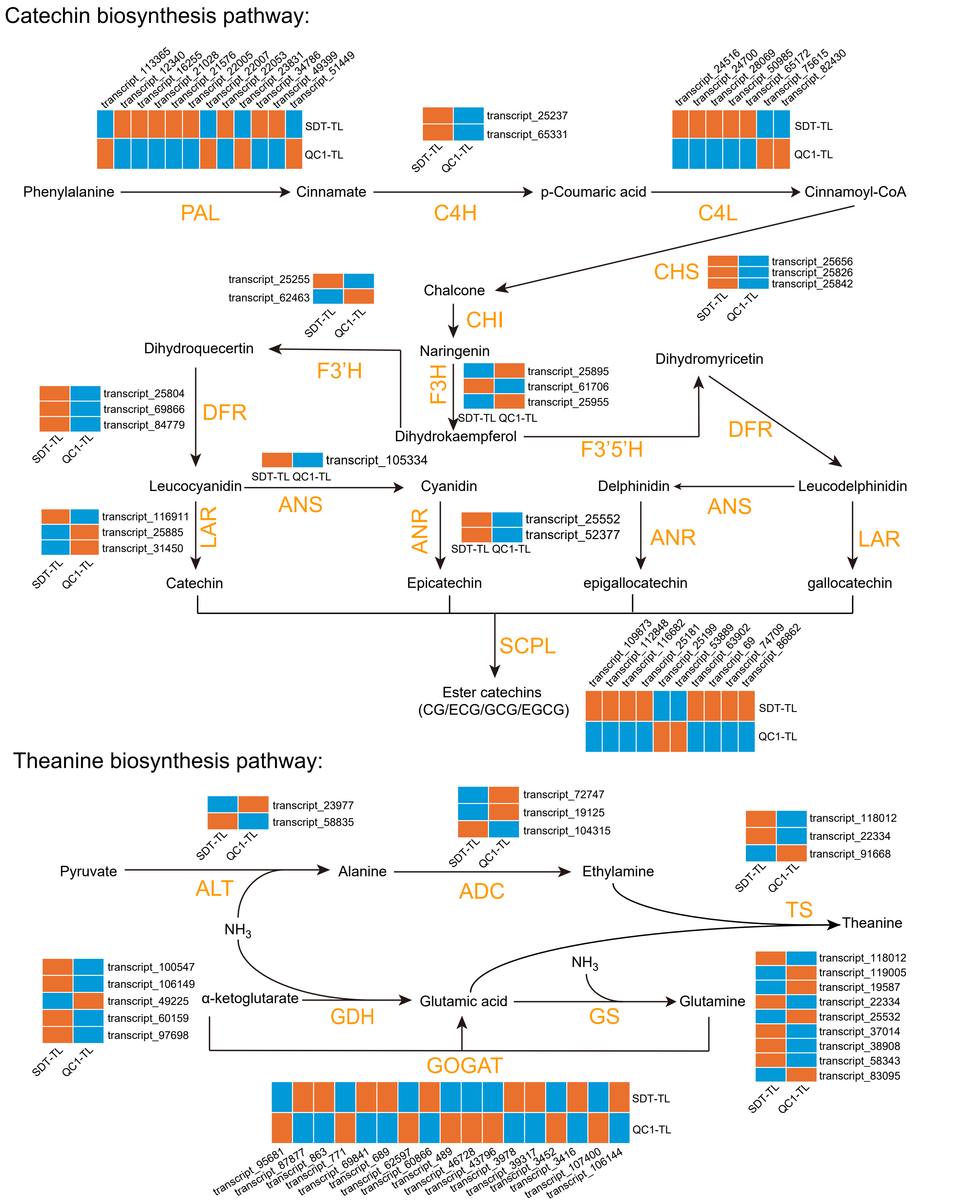

Figure S6.Biosynthetic pathway and gene expression analysis of catechin and theanine metabolism. The orange letters indicate the enzyme associated with the metabolites. Heat map showing the relative expression level of genes in QC1-TL and SDT-TL. Data were the mean values of three biological replicates.
