# Supplementary material for: De novo full length transcriptome analysis of a naturally caffeine-free tea plant reveals specificity in secondary metabolic regulation
Source: Sci Rep. 2023 Apr 12;13:6015. doi: 10.1038/s41598-023-32435-5 (PMC10097665; doi:10.1038/s41598-023-32435-5)
Supplement: Supplementary file 7 — Supplementary Table S1. [file 41598_2023_32435_MOESM7_ESM.docx]

| Samples | Read Number | Base Number | GC Content | ≥Q30 |
| --- | --- | --- | --- | --- |
| QC1-TL1 | 22,938,554 | 6,864,113,184 | 44.93 | 93.18 |
| QC1-TL2 | 19,528,739 | 5,840,491,988 | 44.72 | 93.07 |
| QC1-TL3 | 21,826,821 | 6,533,819,820 | 44.51 | 92.61 |
| SD-ML1 | 20,960,219 | 6,279,954,550 | 45.33 | 92.57 |
| SD-ML2 | 20,714,670 | 6,205,746,192 | 45.21 | 92.34 |
| SD-ML3 | 28,471,094 | 8,509,578,878 | 45.3 | 92.17 |
| SD-R1 | 19,959,007 | 5,976,659,288 | 45.14 | 92.85 |
| SD-R2 | 20,665,674 | 6,183,478,754 | 45.29 | 93.33 |
| SD-R3 | 22,323,251 | 6,681,173,580 | 46.07 | 93.21 |
| SD-S1 | 19,722,719 | 5,893,921,622 | 45.02 | 93.47 |
| SD-S2 | 24,286,552 | 7,247,371,710 | 45.19 | 92.77 |
| SD-S3 | 22,721,503 | 6,791,158,064 | 44.92 | 93.15 |
| SD-TL1 | 24,384,413 | 7,286,768,828 | 45.66 | 92.5 |
| SD-TL2 | 22,008,091 | 6,580,806,834 | 45.32 | 92.48 |
| SD-TL3 | 25,544,718 | 7,621,175,932 | 45.46 | 92.57 |

Table S1. Information of RNA-seq data.
