# Supplementary material for: De novo full length transcriptome analysis of a naturally caffeine-free tea plant reveals specificity in secondary metabolic regulation
Source: Sci Rep. 2023 Apr 12;13:6015. doi: 10.1038/s41598-023-32435-5 (PMC10097665; doi:10.1038/s41598-023-32435-5)
Supplement: Supplementary file 8 — Supplementary Table S2. [file 41598_2023_32435_MOESM8_ESM.docx]

| Gene name | Gene ID | NR annotation | Identity (%) | Species name | Sequence ID |
| --- | --- | --- | --- | --- | --- |
| PAL1 | P01_transcript_51449 | Phenylalanine ammonia lyase | 99.49 | *Camellia sinensis* | XM_028210520.1 |
| PAL2 | P01_transcript_12340 | Phenylalanine ammonia lyase | 99.34 | *Camellia sinensis* | MN207178.1 |
| PAL3 | P01_transcript_22053 | Phenylalanine ammonia lyase | 99.53 | *Camellia sinensis* | MN207173.1 |
| F3H | P01_transcript_25955 | Flavanone 3-hydroxylase | 99.41 | *Camellia sinensis* | XM_028197260.1 |
| ADC1 | P01_transcript_72747 | Arginine decarboxylase | 99.35 | *Camellia sinensis* | XM_028266866.1 |
| ADC2 | P01_transcript_19125 | Arginine decarboxylase | 99.67 | *Camellia sinensis* | XM_028248840.1 |
| GOGAT | P01_transcript_106144 | Glutamate synthase | 98.93 | *Camellia sinensis* | XM_028228546.1 |
| GS1 | P01_transcript_19587 | Glutamine synthetase | 99.49 | *Camellia sinensis* | JQ925872.1 |
| GS2 | P01_transcript_37014 | Glutamine synthetase | 99.54 | *Camellia sinensis* | JQ925872.1 |
| GS3 | P01_transcript_83095 | Glutamine synthetase | 99.57 | *Camellia sinensis* | JQ925872.1 |
| ALT | P01_transcript_23977 | Alanine aminotransferase | 99.35 | *Camellia sinensis* | XM_028267769.1 |
| TCS1 | P01_transcript_65998 | Caffeine synthase | 99.53 | *Camellia sinensis* | AB031280.1 |
| TCS2 | P01_transcript_97158 | Caffeine synthase | 98.80 | *Camellia sinensis* | AB031281.1 |
| ERF1 | P01_transcript_46740 | Ethylene-responsive transcription factor | 97.47 | *Camellia sinensis* | XM_028250704.1 |
| ERF2 | P01_transcript_50094 | Ethylene-responsive transcription factor | 99.06 | *Camellia sinensis* | XM_028242645.1 |
| WRKY | P01_transcript_57872 | WRKY transcription factor | 99.02 | *Camellia sinensis* | XM_028265190.1 |

Table S2. The unigenes related to catechin, caffeine, and theanine metabolic pathway in full-length transcriptome.
